# Proof of Principle for a Real-Time Pathogen Isolation Media Diagnostic: The Use of Laser-Induced Breakdown Spectroscopy to Discriminate Bacterial Pathogens and Antimicrobial-Resistant *Staphylococcus aureus* Strains Grown on Blood Agar

**DOI:** 10.1155/2013/898106

**Published:** 2013-09-10

**Authors:** Rosalie A. Multari, David A. Cremers, Melissa L. Bostian, Joanne M. Dupre, John E. Gustafson

**Affiliations:** ^1^Applied Research Associates, Inc., 4300 San Mateo Boulevard, NE Suite A-220, Albuquerque, NM 87110, USA; ^2^Microbiology Group, Department of Biology, Molecular Biology Program, New Mexico State University, Las Cruces, NM 88003, USA; ^3^Department of Biochemistry and Molecular Biology, Oklahoma State University, Stillwater, OK 74078, USA

## Abstract

Laser-Induced Breakdown Spectroscopy (LIBS) is a rapid, *in situ*, diagnostic technique in which light emissions from a laser plasma formed on the sample are used for analysis allowing automated analysis results to be available in seconds to minutes. This speed of analysis coupled with little or no sample preparation makes LIBS an attractive detection tool. In this study, it is demonstrated that LIBS can be utilized to discriminate both the bacterial species and strains of bacterial colonies grown on blood agar. A discrimination algorithm was created based on multivariate regression analysis of spectral data. The algorithm was deployed on a simulated LIBS instrument system to demonstrate discrimination capability using 6 species. Genetically altered *Staphylococcus aureus* strains grown on BA, including isogenic sets that differed only by the acquisition of mutations that increase fusidic acid or vancomycin resistance, were also discriminated. The algorithm successfully identified all thirteen cultures used in this study in a time period of 2 minutes. This work provides proof of principle for a LIBS instrumentation system that could be developed for the rapid discrimination of bacterial species and strains demonstrating relatively minor genomic alterations using data collected directly from pathogen isolation media.

## 1. Introduction

The goal of this work is to evaluate Laser-Induced Breakdown Spectroscopy (LIBS) as a tool for the rapid discrimination of bacterial cultures. LIBS is of interest for this application because of its speed of analysis, and because standard identification practices cannot easily distinguish all bacterial pathogen colonies. In LIBS, a laser pulse is focused onto a sample to vaporize and excite *μ*g to ng amounts of material and generate a microplasma or laser spark. Light from the spark is collected and directed to a spectrometer to produce a spectrum that is recorded. The spectrum represents a combination of spectral signals from atoms and molecules of the samples and the surrounding atmosphere. Because the microplasma is formed by focused light, typically little to no sample preparation is required and, with automated analysis, results are available within seconds to minutes. LIBS is an analysis technique that is an outgrowth of atomic emission spectroscopy circa 1860 in which samples were placed in a flame and the colors observed were used for analysis [1]. Since these early experiments, plasma excitation sources such as the electrode spark and inductively coupled plasma have been developed. The first report of the use of a laser pulse for spectrochemical excitation was published in 1963 [2]. Since then, characteristics of the laser spark have been well studied, and LIBS has progressed from being a novelty to being a proven analysis technology. LIBS has been applied across a broad range of applications that include industrial processing, environmental monitoring, coal analysis, sorting of metals and plastics, cultural heritage studies, detection of toxic metals in water, detection of explosive, biological, and chemical materials, rock and soil analysis, aerosol analysis, and the detection of trace elements in fresh vegetables and food powders [3–5]. LIBS has reached a state of technological acceptance such that there is even a LIBS instrument operating on the surface of Mars (ChemCam). Most applications rely on analysis of elemental emission lines observed in the LIBS spectra. More recently, advanced chemometric [6] or other analysis techniques have been applied to LIBS spectra for both classification and identification of complex materials in addition to traditional elemental analysis. LIBS has been investigated as a tool for the discrimination of bacterial genera and species strains [7–14]. LIBS has also been used to discriminate viruses [15]. 

Previously, the ability to discriminate bacterial species, using data collected directly from pathogen isolation media using chemometric methods applied to LIBS spectra, has been demonstrated [15]. Here, we demonstrate the use of LIBS to discriminate a much more complex group of bacterial species and strains than what was previously demonstrated using chemometric analysis combined with a method of building LIBS detection algorithms suitable for deployment on LIBS instrumentation [11, 15, 16]. This work differs from previously published work by others in that the analysis does not rely on libraries for comparison, and analysis does not rely on the identification of individual elemental emissions. The analysis presented is the result of treating the LIBS spectra as essentially fingerprints of the bacterial species and strains in colony form on blood agar. Selected for this study are common pathogenic bacterial species (*Acinetobacter baumannii*, *Escherichia coli*, *Klebsiella pneumoniae*, *Pseudomonas aeruginosa*, and *Staphylococcus aureus*),* Bacillus subtilis*, and tightly related *S. aureus* strains grown on blood agar, as well as isogenic *S. aureus* strains that differ only by the acquisition of mutations leading to increased fusidic acid or vancomycin resistance or an engineered plasmid.

## 2. Methods

### 2.1. Bacterial Strain Construction, Characterization, and Preparation for LIBS Analysis

The bacterial strains utilized in the study are described in Table 1. Briefly, *S. aureus* strain SH1000 is a standard wild-type laboratory *S. aureus* used for genetic manipulation, and strain SH1000-1 is a fusidic acid-resistant mutant of SH1000 that was selected off a Mueller-Hinton agar (Difco laboratories) plate containing 2 mg l^−1^ of fusidic acid. Following the selection for fusidic acid resistance, the fusidic acid minimum inhibitory concentrations MICs were determined in standard liquid media as previously described [17]. *S. aureus *strains LP9, MM61, MM66, and MM66-4 have also been previously described [11, 17]. Based on pulsed-field gel electrophoresis chromosomal RFLP analysis, MM61 is highly related to hetero-vancomycin-intermediate *S. aureus* (hVISA) strain MM66 [17] and MM66-4 is a vancomycin-intermediate *S. aureus *(VISA) MM66 mutant [17].

Comparative genomic sequencing (CGS) services provided by Roche NimbleGen Inc. (Madison, WI, USA) were utilized for whole genome mutation mapping and mutant gene resequencing, to compare parent strains MM66 and SH1000 to MM66 VISA mutant MM66-4 and fusidic acid-resistant mutant SH1000-1, respectively. A *S. aureus *tiling array was used to hybridize test (SH1000-1 and MM66-4) and reference (SH1000 and MM66) genomic DNA, and single-nucleotide polymorphisms in each strain were identified based on previously defined criteria [23]. The tiling arrays were made from the genome of *S. aureus* strain COL for testing MM66 against MM66-4 and *S. aureus* strain NCTC8325 for testing SH1000 against SH1000-1.

RN4220 is also a standard wild-type *S. aureus* laboratory strain utilized for genetic manipulation. RN4220-*fai1* was created by electroporating [24] RN4220 with plasmid pCL52.2::*fai1*, which is an *E. coli*-*S. aureus* shuttle vector pCL52.2 [25] containing a *fai1 *(fusidic acid induced 1 or SACOL2347) [26] amplicon cloned into the HindIII-EcoRI site of pCL52.2. The *fai1* gene encodes a putative drug efflux pump whose function is unknown, that is highly upregulated in SH1000 following fusidic acid induction [26]. The *fai1* amplicon was generated by the polymerase chain reaction utilizing SH1000 chromosomal DNA isolated as previously described [27] with *fai1* primers, *fai1*-F (TTACTGTCGGGAATTCGTTGTTCCTGGAATGAACGCTGAAG), and *fai1*-R (GGTAATAAAAAAGCTTATCGATAACCATATTTGGCACCGATACT). This *fai1* amplicon is 2079 bp in size and contains the entire *fai1 *coding region (1932 bp) as well as a 57 bp upstream- and a 90 bp downstream-flanking sequence.

To prepare all bacterial strains for LIBS analysis, bacteria were streaked onto a fresh Luria broth agar (LBA) plate which was allowed to grow overnight (37°C, 18 hr). Single colonies on the LBA plate were then streaked onto a 5% (vol/vol) bovine blood agar (BA) plate which was then allowed to incubate overnight. The next morning, to create a larger surface area of bacterial material for LIBS data collection, the colonies on the BA plates were spread over the entire surface of the BA plate using an ethanol-flame glass hockey stick. 

### 2.2. LIBS Spectra Collection

**Figure 1:**
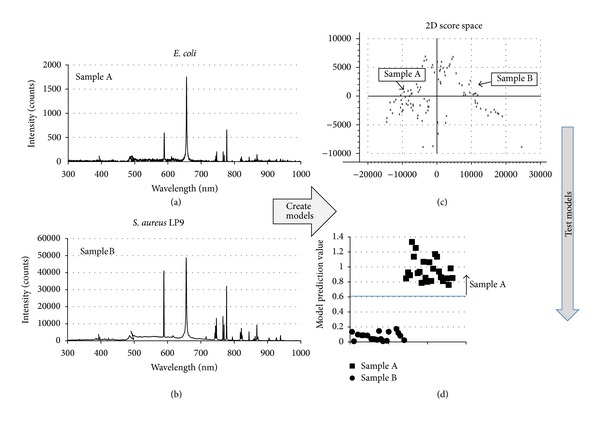
(a), (b) Examples of LIBS spectra used to create a discrimination model for *E. coli* and *S. aureus*. (c) The two-dimensional score space plot for the resulting model showing discrimination between the sample types. (d) The prediction values obtained by testing the model with the verification spectra.

The experimental set-up used for LIBS data collection from the uninoculated BA plates and BA cultures has been described previously [15]. Briefly, pulses from a Q-switched Nd:YAG laser (1064 nm, 60 mJ/pulse, 10 Hz) were focused onto the sample by orienting the open plate end towards the laser and sparking the pathogen covered BA. The laser energy was selected experimentally such that a spark resulting in a LIBS spectrum of strong intensity yet not saturating the detector could be generated on the surface of the BA plate from a distance outside the BSL-2 hood. Data were collected with all BA samples located in a biological safety hood. Plasma light was collected using an off-axis parabolic mirror and fiber optic and then routed to a dual-channel spectrometer/detector system (Avantes AvaSpec-ULS2048-2-USB2). Because the samples were moved around manually in front of the laser beam to target the pathogen on the surface of the BA and ensure a fresh spot for each spectrum, the lens-to-sample distance changed slightly during data collection. A hole in the parabolic mirror permitted the optical path of the laser pulses and light collection to be collinear, eliminating parallax as a result of changes in the sample distance. Each recorded spectrum was the average of ten single-shot spectra (detector acquisition parameters: 1 *μ*sec delay, 1.1 msec window). A total of 100 averaged spectra were collected from each sample. Representative spectra for *E. coli* and *S. aureus* are shown in Figures 1(a) and 1(b). Of the 100 spectra, 50 were used to build the identification models and the remaining 50 were then used to test the models (verification spectra). Other numbers of spectra could have been chosen for calibration and verification spectral groups, but based on previous experience, 50 of each were chosen for this study.

### 2.3. Data Analysis

The data analysis procedure used here has been described previously [11, 15]. Briefly, to discriminate samples or groups of samples, mathematical models were developed and then used in a predictive flow based on sequential screening [28]. The discrimination models are based on single-variable partial least square regression combined with principal component analysis. This technique, referred to as PLS or partial least squares, is especially useful when trying to predict a set of dependent variables from a very large set of independent variables. For this analysis, the dependent variable is the sample and the independent variables associated with the sample are the intensity measurements at each wavelength, that is, the LIBS spectrum, encompassing 4096 channels from 232 to 1026 nm in wavelength. Once a model has been generated for the sample classes, its predictive power is evaluated using the verification spectra collected at the same time as the spectra used to build the models.  Figure 1(c) shows the two-dimensional score space plot for the first two principal components (PC) of the model. Typically, over 80% of the observed variance in the modeling can be explained using just these two components. The result of running the verification spectra through the model is a prediction value (in this case, typically between 0 and 1) used to match the test sample to one of the sample classes. Prediction values outside the range 0 to 1 are possible and indicate some degree of mismatch between the modeled spectra and the verification spectra. For this experiment, the mismatch may be attributed to a fluctuation in the coupling of the laser pulse into sample during data collection. A change in the coupling of the pulse and sample will result in spectral differences affecting the model prediction. However, mismatched spectra can still be classified using this method as all that is important for the classification is whether the prediction value lies above or below the value chosen for differentiation.

**Figure 2:**
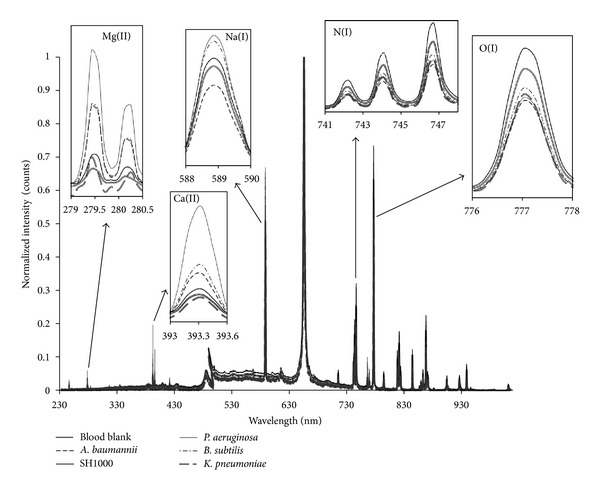
Examples of LIBS classification spectra used for building models for species and blank blood agar differentiation.

**Figure 3:**
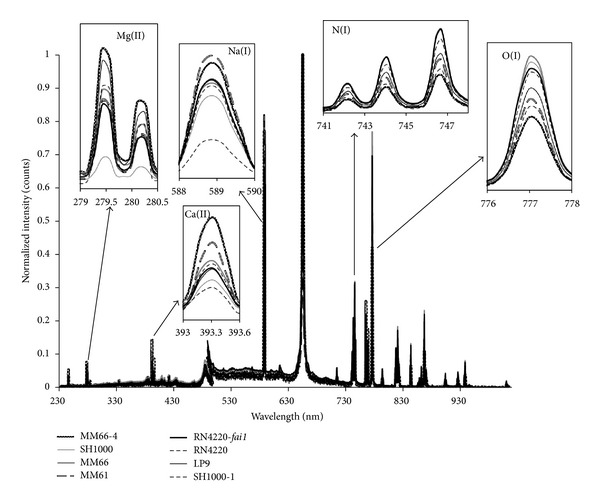
Examples of LIBS classification spectra used for building strain models.

Examples of LIBS classification set spectra, obtained for the samples and used as input to create the PLS1 regression models, are shown in Figure 2 for the various bacterial species investigated and Figure 3 for the *S. aureus* strains. At first, the spectra seem very similar but, on closer inspection, elemental compositional differences can be clearly seen. For all samples, emissions from Mg, Na, N, O, and Ca are observed but there are differences in the spectral intensities for these elements when compared across the sample spectra. These differences in elemental lines and their associated intensities contribute to the creation of a distinctive set of 4096 variables for each sample. 

A good discrimination model is considered to be the one that results in a sufficiently wide separation between the prediction values for the two groups being discriminated such that a line can be drawn above which all prediction values are reliably associated with one sample group. Verification samples with the highest prediction values would be considered matched to the sample being discriminated. Samples with lower prediction values would be considered matched to the samples not being discriminated. Having such a separation is critical to the ability to deploy detection algorithms on LIBS instrumentation.  Figure 1(d) illustrates this process. The best models are those for which there is a wide separation in the prediction values obtained from verification spectra. To improve the observed separation, prediction values from individual spectra were averaged (typically 50 but less in some cases when fewer spectra were available for testing model performance because saturated spectra were excluded from the analysis). Once a good model was created, the model was placed in the algorithm flow, the sample group discriminated was removed from the discrimination process, and the process was repeated to discriminate between the remaining samples until a model was created to discriminate another sample group. This process was repeated until all sample groups were identified to create the overall detection algorithm. 

It should be understood that the type of analysis performed here detects the targeted bacteria within a certain matrix (e.g., agar) and the surrounding atmosphere. The collected LIBS spectra are a combination of signals from all three sources. Changing the isolation media or discrimination across a variety of isolation media requires the development of a new algorithm that incorporates LIBS spectral data from all groups to be discriminated. In addition, the LIBS spectrum is affected by measurement parameters such as laser pulse energy, lens-to-sample distance, and detector timing parameters. For the lens-to-sample distance used here (30 cm), the detector timing parameters (1 *μ*sec delay, 1.1 msec integration period) and pulse energy (60 mJ) were selected to generate a strong recorded spectrum without saturating any emission features. For optimum performance, the values of these parameters, used to record the spectra used for model development should agree with those used to discriminate actual samples. When considering the development of LIBS instrumentation for the discrimination of pathogens using data collected directly from pathogen isolation media, these constraints translate to discrimination algorithms tailored to the instrument and its planned use. In this work, the ability to differentially identify samples within a predefined set of samples (contaminant + matrix) for fixed experimental sampling conditions critical to the successful development of LIBS-based instrumentation is demonstrated. In addition, the methodology presented is useful for developing LIBS instruments for specific applications in which sampling conditions can be fixed, the samples to be discriminated can be characterized, and the natural sample variability can be captured in the detection algorithm. The specific algorithms created in this study are applicable only to the equipment configuration that was used to collect these data and only for the detection in the matrix of blood agar pathogen isolation media selected. However, the detection algorithm development methodology for a different equipment configuration or different pathogens and/or pathogen isolation media would be the same.

## 3. Results

### 3.1. Mutational Characterization of Antimicrobial-Resistant and Susceptible Isogenic Strains of *S. aureus*

As expected, *S. aureus* strain SH1000-1 demonstrated an increased fusidic acid MIC (32 mg/L) compared to that of parent strain SH1000 (0.125 mg/L). Mutations within *fusA* which encodes the target of fusidic acid, elongation factor G, are associated with fusidic acid resistance in *S. aureus* [29]. CGS uncovered a total of 5 nonsynonymous intragenic mutations in SH1000-1 compared to SH1000 (Table 2). One of the SH1000-1 mutations was a nonsynonymous mutation within *fusA *(Table 2), previously determined to convey fusidic acid resistance on *S. aureus* [29]. Four additional mutations affecting 3 codons in a gene encoding a putative phage protein were also detected in SH1000-1, yet, any role these mutations might have on the acquisition of fusidic acid resistance by *S. aureus* needs to be further investigated. 

CGS confirms that MM66-4 contains a total of 8 chromosomal mutations compared to parent strain MM66 (Table 2). One of these mutations that has occurred appears in *yycFG* encodes a two-component regulatory system that controls cell wall autolysis [30, 31], and altered *yycFG* transcription has been implicated in the control of the VISA mechanism [32].

### 3.2. Creation of a Discrimination Algorithm Using Multivariate Regression Analysis

**Figure 4:**
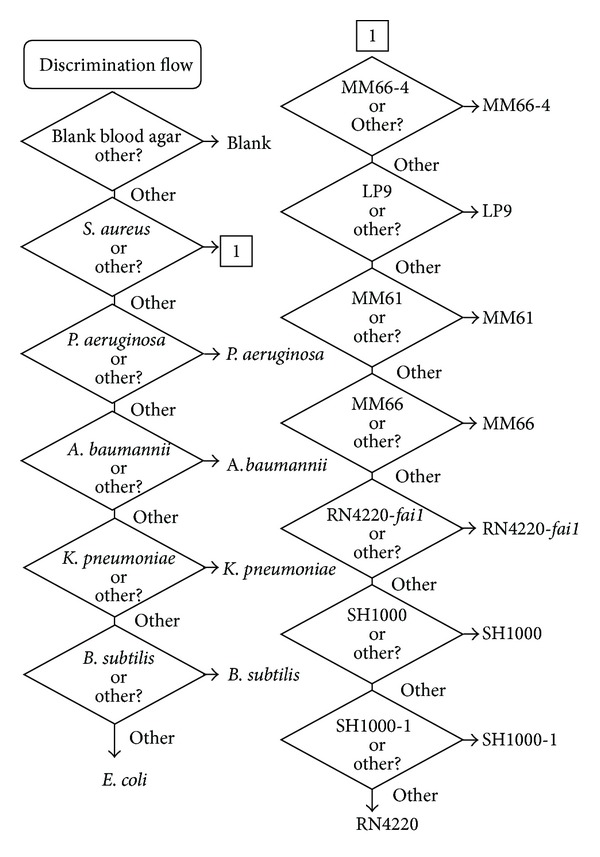
Complete discrimination flow for the discrimination of pathogens on blood agar isolation media.

**Figure 5:**
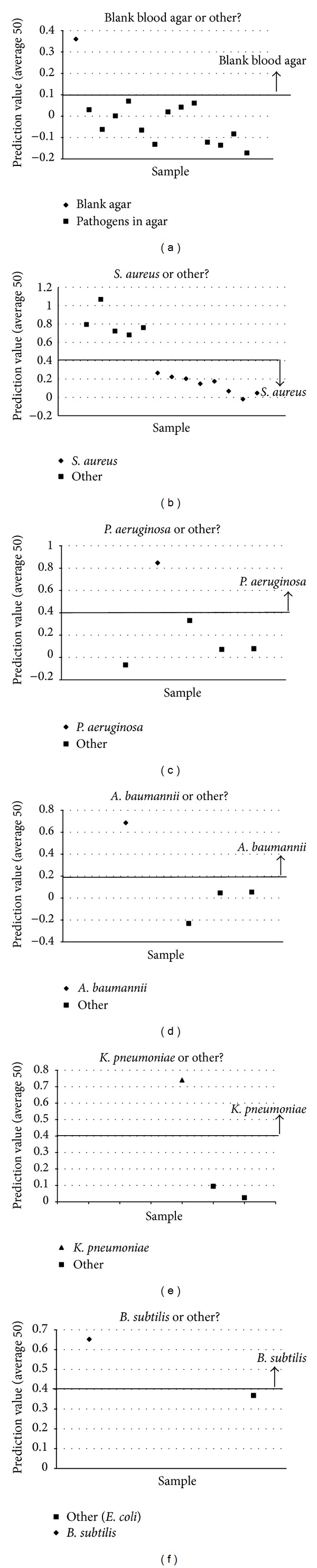
Plots of the prediction values obtained when discrimination models in the main branch of the discrimination algorithm were tested using the verification spectra.

**Figure 6:**
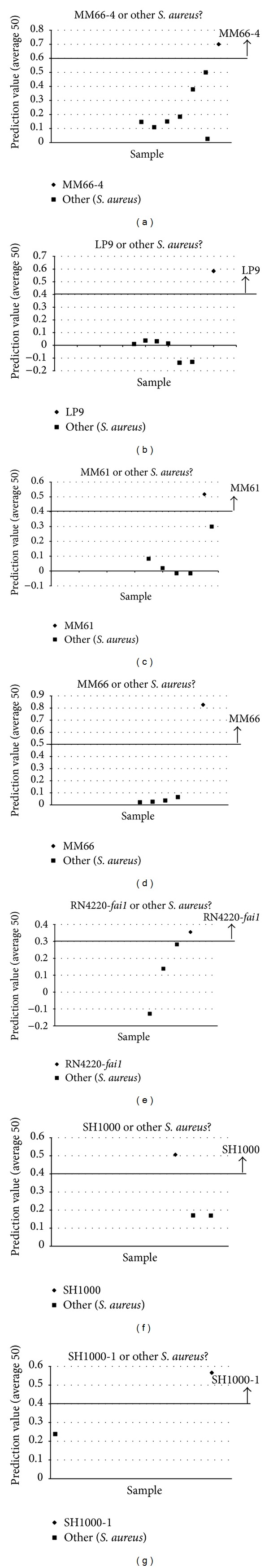
Plots of the prediction values obtained when discrimination models in the *S. aureus* branch of the discrimination algorithm were tested using the verification spectra.

Using the methods described above (discriminating one group of samples, removing it from the analysis, creating a discrimination model to differentiate the next group of samples, and then iterating this process until all samples have been differentiated) it was possible to create a discrimination algorithm capable of correctly identifying all samples included in this study.  Figure 4 shows the algorithm structure. The uninoculated BA (blank) was the easiest sample to discriminate. Once this sample was removed from the discrimination, it was then easier to separate the *S. aureus* BA cultures as a single group to be, subsequently, discriminated separately. After the *S. aureus* samples were removed from the analysis set, the next easiest sample to discriminate was the *P. aeruginosa*, followed by* A. baumannii*, *K. pneumoniae*, *B. subtilis*, and *E. coli*. The most difficult samples to discriminate from the study group were *B. subtilis* and *E. coli* as evidenced by their position at the end of the discrimination flow. See Figure 5 for plots of the prediction values obtained when these models were tested using the verification spectra. For the *S. aureus* sample group, the easiest sample to discriminate from the study group was MM66-4, whereas SH1000-1 and RN4220 were the most difficult to distinguish with the other strains falling in between.  Figure 6 shows plots of the prediction values obtained when these models were tested on the verification spectra.

## 4. Discussion

*S. aureus *and *E. coli* are a major cause of all hospital-borne infections as well as community-acquired infections [33, 34]. *A. baumannii*, *P. aeruginosa*, and* K. pneumoniae* are also major contributors to life-threatening health care-associated community-acquired infections [35–38].

Here, we have demonstrated the ability to use LIBS to discriminate between these bacterial pathogenic species and the model Gram-positive organism *B. subtilis*, from samples grown *in situ*, on the surface of a BA plate*. S. aureus* does not form spores while *B. subtilis* is a spore former and both bacteria are evolutionarily related and phylogenetically placed within the bacterial phyla Firmicutes [39]. *A. baumannii, P. aeruginosa*, *K. pneumoniae*, and *E. coli* are also phylogenetically related and all reside in the bacterial phyla Gammaproteobacteria [39]. Since the use of LIBS for pathogen discrimination relies on elemental emission produced by laser plasma excitation of elements within the living bacterial species and BA growth surface, and not evolutionary relatedness, evolutionary relationships were not reproduced.

All of the pathogens analyzed in this study have been reported to acquire mutation(s) or horizontally transmitted genes that allow them to resist the action of antimicrobials designed to treat infections caused by these pathogens [37, 38, 40–42]. Over the past almost 60 years, vancomycin has remained a therapeutic option for serious infections caused by multiply antimicrobial-resistant methicillin-resistant *Staphylococcus aureus* (MRSA). Vancomycin targets late stage peptidoglycan synthesis in Gram-positive bacteria by binding to the terminal D-alanine-D-alanine residue of the peptidoglycan precursor preventing cell wall synthesis [43, 44]. Since the first VISA isolates were reported in 1997 [45], a steady stream of reports on VISA strains have been appearing in the literature [46]. In general, hVISA express a low-level vancomycin resistance, yet upon exposure to vancomycin, these strains produce select VISA cell subpopulations [17]. The hVISA and VISA phenotype has been attributed to multiple strain-specific mutations, including those in genes affecting peptidoglycan metabolism that lead to the production of a thickened peptidoglycan layer, an attribute associated with the VISA mechanism [46]. MM66-4 is an isogenic VISA MM66 mutant which differs by 8 point mutations compared to parent strain MM66. While it is difficult to determine exactly which of the 8 mutations contribute to the VISA mechanism, we suspect that the mutation in *yycG* might be proven significant to the elevated vancomycin resistance observed in MM66-4 compared to that of MM66.

Fusidic acid is a novel steroid antimicrobial that has been used throughout Europe and Australia and, in combination with rifampicin, provides an option for the treatment of MRSA infections [47]. Fusidic acid interferes with bacterial protein synthesis by preventing the release of the elongation factor G-GDP complex from the ribosome [48]. SH1000-1 is a fusidic acid-resistant mutant of parent strain SH1000 that harbors 5 intragenic mutations, including a mutation previously demonstrated to confer fusidic acid resistance in *fusA*, which encodes the fusidic acid target elongation factor G. 

We have shown that LIBS combined with chemometric analysis can be used to discriminate between our characterized mutation-mediated antibiotic-resistant mutants and parent strains of *S. aureus *grown on BA. The strain pairs discriminated using LIBS include the related vancomycin-susceptible strain MM61 and hVISA strain MM66; MM66 and VISA MM66-4; and fusidic acid-resistant SH1000-1 and SH1000. LIBS analysis also discriminated RN4220 from its transformed isogenic strain RN4220-*fai1*, which harbors pCL52.2::*fai1 *that encodes plasmid functions, harbors a unique fusidic acid inducible gene, and encodes tetracycline resistance. 

We speculate that the unique genetic alterations that discriminate MM66 from MM61, MM66 from MM66-4, SH1000 from SH1000-1, and RN4220 from RN4220-*fai1* lead to enough adjustment in the cellular elemental composition and/or possibly, the ability to degrade the blood in BA, that LIBS analysis can now discriminate these tightly related strains grown on BA. It is possible that an alteration in the overall peptidoglycan structure and metabolism in MM61 compared to MM66 and MM66 compared to MM66-4 contributes to the elemental alterations that allow for LIBS discrimination of these strains.

Determining both bacterial pathogen identity and antimicrobial resistance phenotype, as fast as possible, is imperative when determining which antimicrobial regimen will best suit a patient suffering from an infection caused by a bacterial pathogen. This work adds to the growing LIBS-bacterial pathogen discrimination literature, by demonstrating that LIBS technology can be used to discriminate bacterial species grown on BA. It also demonstrates the potential of LIBS technology to rapidly identify antimicrobial-resistant bacteria from susceptible organisms *in situ* following the growth on BA.

## Figures and Tables

**Table 1:** Bacterial species and strains utilized in this study.

Species	Species	Relevant characteristics	Reference
*A. baumannii *	ATCC BAA-1789	Multiple antibiotic-resistant	http://www.atcc.org/
*B. subtilis *	ATCC 23857		[18]
*E. coli* K12	ATCC 10798		[19]
*K. pneumoniae *	ATCC 13882		[20]
*P. aeruginosa *	ATCC 3350		http://www.atcc.org/
*S. aureus *	SH1000	Standard laboratory strain	[21]
*S. aureus *	SH1000-1	Fusidic acid-resistant SH1000 strain	This study
*S. aureus *	RN4220	Standard laboratory strain	[22]
*S. aureus *	RN4220-*fai1 *	RN4220 with plasmid pCL52.2::*fai1 *	This study
*S. aureus *	LP9	Clinical MRSA	[17]
*S. aureus *	MM61	Clinical MRSA	[17]
*S. aureus *	MM66	Clinical MRSA	[17]
*S. aureus *	MM66-4	Laboratory-derived MM66 VISA mutant	[17]

**Table 2:** Mutations detected in MM66 and SH1000-1 by comparative genome sequencing.

Strains	SACOL loci^a^	Gene	Function	SNP position^a^	Amino acid change^a^
SH1000-1	*Intragenic *				
	SACOL0593	*fusA *	Elongation factor G	C^617228^→ T^617228^	H^457^→ Y^457^
				
	SACOL0358			A^371671^→ T^371671^	N^36^→ I^36^
				T^317672^→ A^371672^	N^36 ^→ I^36^
				G^371676^→ A^371676^	E^37^→ K^37^
				A^371685^→ C^371685^	K^40^→ Q^40^

MM66-4	*Intragenic *				
	SACOL1883-		Hypothetical protein	C^1938819^→ T^1938819^	
	TRNA-ser		tRNA-ser	T^1938825 ^→ A^1938825^	
				
	SACOL1947-		Hypothetical protein	C^2010604^→ T^2010604^	
	SACOL1948		Hypothetical protein	A^2010605^→ G^2010605^	
				
	SACOL2575-		Putative aromatic Amino transferase	A^2638759^→ T^2638759^	
	SACOL2576	*crtN *	Squalene synthase	C^2638762^→A^2638762^	
	*Intragenic *				
	SACOL1690	*apt *	Adenine phosphoribosyl- transferase	C^1721075 ^→T^1721075^	A^57^→ V^57^
	SACOL0020	*yycG *	Sensory box histidine kinase	A^26449 ^→ G^26449^	K^263^→ E^263^

SNP: single nucleotide polymorphism.

^a^Based on loci numbers, nucleotide positions, and amino acid residues in NCBI Genbank database *S. aureus* strain COL reference genome.
